# Physical forces modulate cell differentiation and proliferation processes

**DOI:** 10.1111/jcmm.13417

**Published:** 2017-11-30

**Authors:** Laurent Schwartz, Jorgelindo da Veiga Moreira, Mario Jolicoeur

**Affiliations:** ^1^ Assistance Publique des Hôpitaux de Paris Paris France; ^2^ LIX ‐ UMR 7161 Campus de l’École Polytechnique Palaiseau France; ^3^ Research Laboratory in Applied Metabolic Engineering Department of Chemical Engineering École Polytechnique de Montréal Montréal QC Canada

**Keywords:** paradigm shift, biology, gene expression, mitochondria, mechanical stress, electrical potentials, thermodynamics

## Abstract

Currently, the predominant hypothesis explains cellular differentiation and behaviour as an essentially genetically driven intracellular process, suggesting a gene‐centrism paradigm. However, although many living species genetic has now been described, there is still a large gap between the genetic information interpretation and cell behaviour prediction. Indeed, the physical mechanisms underlying the cell differentiation and proliferation, which are now known or suspected to guide such as the flow of energy through cells and tissues, have been often overlooked. We thus here propose a complementary conceptual framework towards the development of an energy‐oriented classification of cell properties, that is, a mitochondria‐centrism hypothesis based on physical forces‐driven principles. A literature review on the physical–biological interactions in a number of various biological processes is analysed from the point of view of the fluid and solid mechanics, electricity and thermodynamics. There is consistent evidence that physical forces control cell proliferation and differentiation. We propose that physical forces interfere with the cell metabolism mostly at the level of the mitochondria, which in turn control gene expression. The present perspective points towards a paradigm shift complement in biology.

**Table:** 

**• Introduction**
**• Cells sense mechanical and osmotic forces**
**• Electromagnetic forces affect cell fate**
**• Metabolism is written as a binary code (anabolism/catabolism)**
**• The mitochondria, a control switch of proliferation and differentiation**
**• The mitochondria controls gene expression**
**• Physical forces control the mitochondria**
**• Conclusion**
**• Conflict of interest**
**• Acknowledgement**

## Introduction

The formation of cell membranes has conferred a main advantage to the original cells by enabling metabolic homoeostasis 1, 2, with the occurrence of the possibility to maintain cell viability 3. While developed from and within cell‐environment interactive phenomena, the cells, as defined entities, were competent to continuously find ways to adapt to its changing environment and thus to evolve. From the primitive life organization within a cell entity, surrounding forces have played a primary role in cell evolution. A plethora of mechanisms developed along with cell evolution allow a cell to continuously sense the quality of its environment, signalling the activation and/or the inhibition of some specific metabolic processes enabling the maintain of cell viability, or in some cases routing the cell towards dormancy (*e.g*. bacterial or fungal sporulation) or cell death through programmed sacrifice (*i.e*. apoptosis) or non‐controlled (*i.e*. necrosis and cell lysis) mechanisms 4, 5, 6.

With a cytoskeleton connecting its membrane to nucleus DNA 7, 8, a cell also senses physical forces such as osmotic 4, mechanic 9, electric and magnetic 10, 11, 12, 13. Indeed, this sensing capacity is particularly important to the development, and the maintain of organized eukaryotic cells, such as within humans, and the laws of physics are known to play a key role in medicine. Traction and pressure are key parameters in orthopaedics, as well as in the Starling law in cardiology 14, 15. However, biology has often lost in sight the influence of physics laws that play a crucial role in governing the transformation of energy in both matter and living systems. Energy, the capacity of the system to perform work, takes many forms in biological systems. At the cellular level, biological functions are primarily regarded as being influenced by chemical, electric and mechanical energies. The physical and chemical sciences thus provide the foundation for physiology, and we may expect biology recognizing that the processes of life are mechanistically determined by physico–chemical forces 1, 16, 17, 18, 19, 20. We thus here put forward a conceptual framework that outlines an integrative approach to classify cell speciation based on physics‐based phenotypes. The same laws that govern inorganic and organic matter are considered.

To take an example, the growth and maturation of the hand are a highly predictable phenomenon 21, 22, 23. During embryogenesis, bones of the diaphysis are formed on an initial cartilaginous model. Cartilage is later replaced by bone; this process is called enchondral ossification 21, 24. Bone development is so predictable that a simple radiological picture of the right hand is used to confirm the age of the infant 21. The proper development of the musculoskeletal system requires the co‐ordinated development of cartilage, bone, muscle and tendon. In the embryo, ossification of the cartilaginous anlagen of the metatarsus starts in parallel with active movement of the feet by muscle contraction. Mechanical stress resulting from muscle contraction seems to guide enchondral ossification patterns. Only the simple law of physics could guide such a process 24.

## Cells sense mechanical and osmotic forces

**Figure 1:**
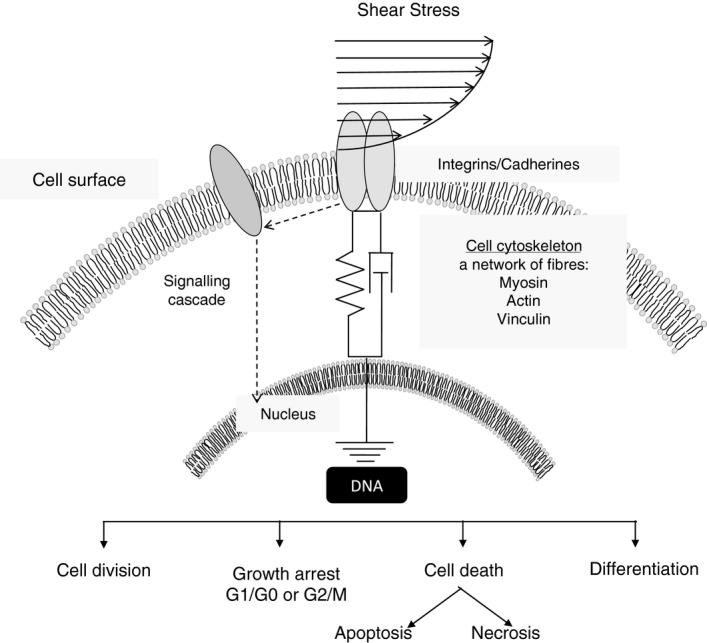
Mechano‐transduction intracellular signalling. Adapted from Chang *et al*., 2008; Ingber 2006; Wang *et al*., 2009.

The influence of mechanical energy on living organisms is omnipresent. Cells are continuously subjected to stretching, compression and shear forces that influence cell division, gene expression, cell migration, morphogenesis, cell adhesion, fluid homoeostasis, ion channel gating and vesicular transport (Fig. 1) 8, 25, 26, 27, 28, 29, 30, 31, 32, 33. The seminal work of D'Arcy Thompson demonstrated that mechanical forces play a key role in plant and animal morphogenesis 34. These physical forces displace the relative locations of molecules within cells and tissues, which give rise to viscoelastic deformation of membranes and cytoskeletal and extracellular matrices 25. We already have an intuitive understanding of the distribution of mechanical forces when we consider pressure, which depends not only on environmental and endogenous loads (pressure exerted by cavities and blood) but also on intrinsic mechanical factors of organs, such as shape, architecture and mechanical properties of tissues.

The development of an organ is constrained by internal and external limitations 3. The effect of shear stress on the endothelial cell is the best‐studied example. In addition to oxygen and lactate gradients, shear stress is responsible for vascular network formation, the fractal organization of the arterial and venous trees, as well as the unavoidable tropism of arteries towards capillaries and then veins 35, 36, 37. Branching morphogenesis, a frequent pattern in gland embryogenesis, can also be explained by shear stress gradients 38.

At the cellular level, increased osmotic pressure inhibits cell proliferation 39, 40, 41, 42, 43. In *in vitro* culture, hyperosmotic condition leads to reduced specific growth rate but higher glucose specific uptake rate (*q*_*GLC*_) and lactate specific production rate (*q*_*LAC*_), with a constant ratio (*i.e. q*_*LAC/*_ *q*_*GLC*_), and increased specific recombinant protein production rate *in vitro* 44.

**Figure 2:**
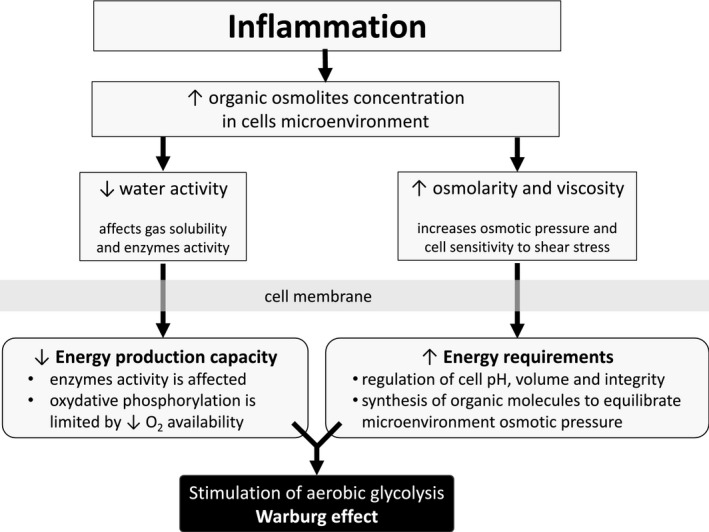
Inflammation results in a high osmolarity environment affecting mitochondrial behaviour.

Increasing osmotic pressure causes a decrease in oxygen solubility, because of a decrease in water activity, a phenomenon directly affecting the cell energetic productivity, which in turn affects a cell capacity to face a change of osmotic pressure 45. In addition, decreasing water activity will affect the enzyme‐substrate complex dynamics as the enzyme properties can be affected in charged electrolytes. A schematic representation of the effect of osmotic pressure on cell behaviour is illustrated in Fig. 2. The mechanisms involved in the change from oxidative phosphorylation to aerobic glycolysis are obviously complex and multifactorial. However, the occurrence of inflammation induces physical forces that specifically affect cell metabolism, limiting the cell energetic production capacity while increasing energetic demand. This energetic deregulation effect favours the activation of aerobic glycolysis to provide the required ATP turnover rate. This situation has been observed in various diseases enumerated in this review such as in cancer 46. Furthermore, atmospheric pressure will affect molecular distance, another phenomenon that can surely play on cell metabolism, also affecting the enzyme‐substrate complex establishment and efficiency 47.

Mesenchymal stem cells are multipotent cells that can be induced to differentiate into a variety of mesenchymal tissues, including bone, cartilage, tendon, fat, bone marrow stroma and muscle. Traction appears to generate condensation and maturation of chondrocytes or feather, scale and hair formation 24. Chondrocytes are known to sense and respond to the mechanical stimuli by multiple regulatory pathways: upstream signalling transcription, translation, post‐translational modifications and vesicular transport 48. Fluid‐induced shear causes chondrocytes to elongate and align 49. Chondrocytes respond to shear stress by an increased secretion of extracellular matrix namely collagen and proteoglycan 48, as well as the modification of metabolism 50, a phenotype that has also been reported in *in vitro* culture of chondrocytes 51.

Similarly, traction induces the secretion of extracellular matrix by fibroblasts, distorts collagen gels and creates patterns that are similar to tendon 52. This morphogenetic rearrangement of extracellular matrix is the primary function of fibroblast traction and explains its excessive strength 52.

Stem cells may be expanded in culture and subsequently permitted to differentiate into the desired lineage. This directed differentiation might be reached by the application of bioactive molecules, growth factors and signalling molecules 52. It is known that physical stress induces the secretion of these growth factors and signalling molecules 38. The question is whether mechanics alone during normal development are sufficient to explain the growth and differentiation of the tissues. If this hypothesis is proven correct, the next question is whether the effects of mechanical forces are being mediated or mimicked through the release of chemicals.

## Electromagnetic forces affect cell fate

Local electric fields within cells result mostly from the motion of charged particles, such as ions Na^+^, K^+^, Ca^2+^ and Cl^−^, across phospholipid bilayer membranes by the opening and closing of channels, as well as active (*i.e*. energetically costly) transporters. This particle motion results from diffusion and electrostatic forces that generate ion gradients and electrochemical potentials. The first cell studied from the point of view of the electricity was the neuron. The flow of electrical currents through an axon was firstly described by the cable theory, developed in the nineteenth century by Lord Kelvin to explain the flow of electricity in submarine cables. Cole and Curtis 53, and Goldman 54, adapted cable theory in the 1920–1940s, considering the resistances and capacitances of the cell membrane and the properties of the electrolytes that surround it. Later, Hodgkin and Huxley considered the influence of ion channels and ionic dynamics to study the electrical conduction and excitability 55.

Electrical currents propagate along the axon in neuronal networks but also play an important role in co‐ordinating the contraction of the heart. Cardiac electrical potentials are generated by the sinoatrial node, the natural pacemaker of the heart, which propagates from the atria to the ventricles *via* the atrioventricular node. Cardiac and skeletal muscle cells are excitable fibre conductors like neurons. In these cells, action potentials are triggered by the arrival of synaptic currents at the neuromuscular junction.

The role of electromagnetic field is not limited to the neuron. Differentiated cells are polarized. Patch clamp technique demonstrates higher transmembrane potentials in differentiated cardiocytes 56. Differentiated cells display a more developed and functional mitochondrial network and rely heavily on oxidative phosphorylation and increased ATP synthesis 57. Ionic gradients and the resulting electrical fields are a direct consequence of oxidative phosphorylation 58. Furthermore, as the cell will work maintaining its membrane electronegativity as well as an intracellular charge equilibrium, local currents of cations and anions generated across the membrane vary along the membrane surface. In addition, electromagnetic fields can affect both macromolecules charge distribution as well as spatial position within a cell volume. This phenomenon can result in elevated and opposite electric fields that can be sensed locally within a cell.

The question is whether these electrical fields are a consequence or a cause for cell differentiation. There are multiple evidence that electrical forces might be responsible for cell differentiation and thus seen as specific effectors initiating and driving cell phenotype evolution. External electrical fields induce cell differentiation 59, 60. Similar results were reported in various works 10, 61, 62. As most molecules in the cells are charged, electrical fields have a direct impact on most cellular functions such as separation of DNA 63, gene expression 64, protein synthesis 65 or even ATP content 66.

## Metabolism is written as a binary code (anabolism/catabolism)

Eukaryote cells, such as prokaryotes, exhibit two opposite metabolisms: anabolic reactions, which consist in biomass synthesis, and catabolic reactions, leading to the breakdown of macromolecules for energetic use, in parallel to constituting a pool of building bloc precursors feeding anabolism 67. Cells convert energy by means of an electron–proton transfer process. The energy of electron flow is conserved in the form of free energy, storage within the ATP, which is used to enable the mechanical, osmotic and biosynthetic work of cells 58. The standard energy of ATP hydrolysis remains within a narrow range among cells with widely varying membrane potential and mechanisms of energy production 68. Oxidative phosphorylation (OXPHOS) provides about 88% of the total energy and substrate phosphorylation (mainly glycolysis) contributes the remaining 12%. In OXPHOS, which occurs within the mitochondria, electrical charges are transferred to oxygen *via* redox reactions, and protons are pumped from the matrix across the mitochondrial inner membrane. ATP is synthesized when protons return to the mitochondrial matrix down their electrochemical gradient. The rate of energy production in OXPHOS is determined by the conductance of the mitochondrial membrane and the electromotive potential across the membrane 69. Energy production in glycolysis, however, is dependent from electrical gradients as these will limit or support mitochondrial activity, which level will result in ATP‐to‐AMP and ATP‐to‐ADP ratios that are known to control the glycolytic rate 70.

Oxidative phosphorylation results in combustion and ATP synthesis, which is resulting in higher transmembrane gradients and therefore increased electrical gradients. Decreased oxidative phosphorylation results in decreased gradients, such as seen in cancer and increased cell proliferation also observed particularly in cancer cells 57.

## The mitochondria, a control switch of proliferation and differentiation

Proliferating cells must, at the end of the cycle, double their biomass (proteins, lipids and nucleic acids) through the cell cycle to result in two (identical) daughter cells. For that, they use the central carbon metabolism (CCM), universally shared among living systems. The CCM is governed by pivotal metabolic pathways such as glycolysis, the pentose phosphate pathway and the citric acid cycle. The cell decision‐making to enter one of these pathways is coupled to redox transitions following nutrient availability. Experimental studies show that the mitochondrial activity is reduced during early progression in the cell cycle in G_1_ 57. Also, the G_1_ phase of the cell cycle is characterized by an anabolic demand in protein synthesis, required for DNA replication in S phase. Synthesis of building blocks, such as amino acids and nucleic acids or pyruvate, from carbohydrate pathways is then a necessary step for biomass synthesis and energy supply through mitochondrial activity.

Mitochondria seem to be more than just an efficient power plant for ATP turnover 71. They are at the core of eukaryotic cell metabolism and cell cycle progression. In there, the tricarboxylic acid (TCA) cycle, branched to glycolysis and to the pentose phosphate pathway, is central supporting mitochondrial metabolism and has been reported to match mitosis.

Mitochondrial biogenesis and metabolic shifts are early events in multiple stem cell differentiation models, with most changes observed in the first stage of the differentiation process. Maturation of the mitochondrial network, as well as increased transcription of mtDNA, is observed during the differentiation of hESCs into cardiomyocytes 72. The development of the mitochondrial network precedes the loss of the pluripotency markers, *OCT4* and *Nanog*, in differentiating hESCs 73. Mitochondrial biogenesis and metabolic shift towards OXPHOS are also early events in osteogenic adipogenic and hepatogenic differentiation. Mitochondrial biogenesis was demonstrated to parallel the loss of pluripotency 74. Along the same lines, it demonstrates that the mitochondrial energy yield controls murine erythroleukaemia cell differentiation 75. In CHO cells, a model simulation study supports the central role of a cell energetic status, in terms of the AMP‐to‐ATP ratio, on the cell central carbon metabolism robustness to a pO_2_ perturbation 70. For instance, T‐cell maturation involves the progression from aerobic glycolysis to OXPHOS 76, 77. The mitochondria DNA has 37 genes all dedicated to mitochondrial functions, which suggests that this energy power plant plays a pivotal decisional role modulating metabolic steady states.

## The mitochondria controls gene expression

While it is widely accepted that the co‐ordination of genetic circuits with developmental bioenergetics is critical to phenotype specification, the metabolic mechanisms that drive cell differentiation are only partially deciphered 78, 79, 80, 81, 82, 83. For the cells to differentiate, anaerobic glycolytic metabolism, while sufficient for embryonic stem cell homoeostasis, must be transformed into the more efficient mitochondrial oxidative metabolism. For example, increasing the mitochondrial efficacy results in cardiocytes differentiation 78.

Mitochondrial activity results in acidification of the cytoplasm in cancer cells 57. Differentiated quiescent cells have a lower pH than proliferating cells 50. Pouyssegur's group showed that the cell cannot proliferate when the intracellular pH is below 7.2 84. Moreover, it has been demonstrated that intracellular pH drives protein synthesis and DNA replication 85, 86, 87. The intracellular acidic pH is followed by global histone deacetylation, leading to chromatin compaction, the phenotype of a dormant cell, like a myocyte or a neuron. Conversely, the intracellular pH increase towards alkalinization is reported to favour acetylation of histone, leading to chromatin decompaction and DNA replication 88, 89.

## Physical forces control the mitochondria

The different forms of energy are interconvertible. The first law of thermodynamics is a statement regarding the conservation of energy: although energy can be converted from one form to another, the total energy of a closed isolated system is constant. This conservation law applies to both inanimate matter and living organisms 90. According to this model, biology could be explained by inefficiency with which cells extract energy from the environment and dynamically distribute this energy throughout the various units that compose the living systems 91. Consequently, mechanical, electric and metabolic energies are intertwined in cells. We recently demonstrated that inflammation, whatever its cause, is synonymous of increased extracellular osmolarity 24, 92, 93, 94, 95, 96, 97.

Increased osmotic pressure results in a transient Warburg effect, a partial and reversible inhibition of the mitochondrial activity 46, 98. The sequence and list of the effects of a high osmotic environment on cell behaviour are illustrated in Fig. 2. The effect of mechanical forces on mitochondria has been poorly studied. This organelle is composed essentially of soft bilayer membranes and many of its functions involve the manipulation of its curvature, as it is easy to sustain curvature strains in a membrane due to its high elasticity. Differences in tension between the two membrane interfaces can create changes in curvature with the displacement of lipids, channels and pumps. As a consequence, the resultant of the electrical forces across curved membranes can change 99. This phenomenon is called flexoelectric effect 99, 100 and it explains, for example, how mechanosensing organelles of hair cells respond to the fluid motion in the inner ear, converting membrane deformation into electric signals. Conformational changes induced by cytoskeletal tension or osmotic pressure may convert mitochondria to a non‐energized state, impairing electrical currents, but allowing mitochondrial smooth movements of fission and fusion. The fact that energized mitochondria have inner membranes extensively curved 101, 102, 103 is quite indicative of a possible role for the flexoelectricity in the energy transformation. Indeed, thermodynamic laws have predicted that membrane tension modulates transmembrane voltage 104 and that a curved membrane maximizes or modulates the organelle interfacial‐to‐volume ratio, such as accumulated cells and capacitors.

## Conclusion

There is overwhelming data demonstrating both the key role of genes and of physical forces (pressure and electromagnetic) in the multiplication and differentiation of cells and on the specific role of physical forces alone. Cells have evolved while being challenged from its close microenvironment, so it is more than probable that these surrounding forces play a direct role in the control of the cellular metabolism by the mean of the mitochondria, which is too crucial for cell survival for not being at the forefront of a cell reaction. Further studies are obviously needed to confirm/infirm this hypothesis.

## Conflict of interest

The authors confirm that there is no conflict of interests.

## Acknowledgement

L.S. initiated the work and analysed the literature together with M.J., who built the figures. All authors have contributed to the literature review and have drafted the manuscript. All authors read and approved the manuscript.
